# A review of functional pancreatic neuroendocrine tumors: Exploring the molecular pathogenesis, diagnosis and treatment

**DOI:** 10.1097/MD.0000000000036094

**Published:** 2023-11-17

**Authors:** Yasir Alshareefy, Sinead Cummins, Adele Mazzoleni, Vidushi Sharma, Saibaba Guggilapu, Amanda Weng Yee Leong, Andrew Awuah Wireko

**Affiliations:** a School of Medicine, Trinity College Dublin, The University of Dublin, Ireland; b Barts and the London School of Medicine and Dentistry, London, United Kingdom; c House Surgeon, Bangalore Medical College and Research; d University of Malaysia, Kuala Lumpur, Malaysia; e Sumy State University, Sumy, Ukraine.

## Abstract

Pancreatic neuroendocrine tumors (PanNETs) are a rare subtype of pancreatic cancer and can be divided into functional (30–40%) and nonfunctional subtypes. The different subtypes of functional PanNETs (F-PanNETs) have a variety of classical presentations that raise suspicion for an underlying PanNET. It is estimated that 90% of PanNETs are sporadic, and the PI3K-Akt-mTOR and ATRX/DAXX signaling pathways have been recognized as key genetic pathways implicated in the pathogenesis. The other 10% of PanNETs may occur in the context of familial cancer syndromes such as MEN1. Chromogranin A is the most useful biomarker currently; however, several studies have shown limitations with its use, especially its prognostic value. Synaptophysin is a novel biomarker which has shown promising preliminary results however its use clinically has yet to be established. Blood tests assessing hormone levels, cross-sectional imaging, and endoscopic ultrasound remain at the core of establishing a diagnosis of F-PanNET. The treatment options for F-PanNETs include surgical methods such as enucleation, systemic therapies like chemotherapy and novel targeted therapies such as everolimus. The prognosis for F-PanNETs is more favorable than for nonfunctional PanNETs, however metastatic disease is associated with poor survival outcomes. Researchers should also focus their efforts on identifying novel pathways implicated in the pathogenesis of F-PanNETs in order to develop new targeted therapies that may reduce the need for surgical intervention and on the establishment of novel biomarkers that may reduce the need for invasive testing and allow for earlier detection of F-PanNETs.

## 1. Introduction

Pancreatic neuroendocrine tumors (PanNETs) are a rare subtype of pancreatic cancer, with an incidence of <1 per 100,000 people according to European and Asian epidemiological studies.^[1]^ PanNETs can be divided into functional and nonfunctional subtypes according to whether or not the neoplasm secretes hormones.^[2]^ There are a variety of subtypes of functional pancreatic neuroendocrine tumors (F-PanNETs) including extremely rare types such as GRFoma, however this review will focus on glucagonomas, insulinomas, VIPomas, gastrinomas, and somatostatinomas, each named according to the hormone they secrete, which account for 30% to 40% of PanNETs.^[3]^ Blood tests assessing levels of these hormones, along with cross-sectional imaging techniques and imaging-guided biopsy for histopathological analysis, remain at the core of establishing a diagnosis of F-PanNETs. Our understanding of the etiology and pathogenesis of this rare group of tumors has been greatly enhanced in recent times, and thus more modern treatment options such as targeted therapies have become available. However, a key challenge in this clinical context is to establish an early diagnosis as metastatic disease is associated with a poor prognosis and 5-year survival rates. As such, our paper aims to explore and summarize the molecular pathogenesis of F-PanNETs, including their genetic origins, current methods of diagnosis, and the management of this heterogeneous group of neoplasms, and expose gaps in the literature where researchers should focus their efforts. There have been numerous papers published in recent times exploring PanNETs as a whole; however, this review is one of the first to focus solely on F-PanNETs and will serve as an important source of education for clinicians on this rare topic.

## 2. Methods

A literature search was conducted on Pubmed, EMBASE, and Web of Science databases using terms for pancreatic neuroendocrine tumors (insulinoma, gastrinoma, etc), molecular pathogenesis, diagnosis, treatment, and prognosis from inception to 2023. The gray literature search was performed using Google Scholar. Articles were only included for the purposes of this review if they were in the English language and their full text was available. The papers retrieved were of a quantitative nature, and their quality assessment was conducted using the Cochrane risk of bias tool.

## 3. Clinical presentation

PanNET is an umbrella term encompassing many subtypes of cancer. They can be divided into functional and nonfunctional tumors. Functional tumors manifest symptoms, and nonfunctional tumors are usually asymptomatic. F-PanNETs produce hormones, which are responsible for their clinical manifestations. Some examples of functional tumors are insulinoma and glucagonoma, which have highly variable presentations due to the physiological actions of these hormones.

### 3.1. Insulinoma

One of the most characteristic findings of insulinoma is fasting hypoglycemia. Placzkowski et al reported, in 2009, that 73% of patients experienced hypoglycemia in a fasting state. 21% of the patients experience hypoglycemia in the fasting and postprandial states, and 6% exclusively postprandially. An increasing number of patients experience only postprandial hypoglycemia symptoms, which are more commonly seen in men.^[4,5]^

Hypoglycemia-related symptoms can be classified as neurogenic or neuroglycopenic. Neuroglycopenic signs and symptoms are those caused by a direct lack of glucose in the central nervous system. Changes in behavior, confusion, exhaustion, seizures, coma, and the possibility of death if nothing is done right away are some of these. Neurogenic signs and symptoms can be either cholinergic (such as hunger, diaphoresis, or paresthesias) or adrenergic (such as tremors, palpitations, or anxiety). In response to what appears to be hypoglycemia, sympathoadrenal involvement (either the release of acetylcholine or norepinephrine) causes neurogenic symptoms and signs.^[5,6]^

Psychiatric symptoms like panic attacks may also be due to insulinoma; however, this is extremely rare.^[5,7]^

### 3.2. Gastrinoma

Abdominal pain and persistent diarrhea are the most prevalent clinical manifestations of gastrinoma. Dyspepsia, gastroesophageal reflux, gastrointestinal bleeding, and weight loss are additional manifestations. A diagnosis of gastrinoma must be taken into consideration in patients who have diarrhea and recurrent or refractory peptic ulcers, particularly multiple and duodenal. It is important to note that only about 0.1% to 1% of patients with peptic ulcer disease have Zollinger-Ellison Syndrome (ZES) as the underlying cause, so widespread screening is not recommended. Proton pump inhibitors, as is typical, improve diarrhea in ZES patients.^[8]^ One of the commonest symptoms of gastrinoma is jaundice. Some of the rarer ways gastrinoma presents is as peptic ulceration or frank ZES.^[9]^

### 3.3. Glucagonoma

Glucagonoma presents with a characteristic rash known as necrolytic migratory erythema (NME), painful glossitis, cheilitis, and stomatitis, weight loss, anemia, hypoaminoacidemia, low zinc levels, deep vein thrombosis, and depression.^[10]^ NME is a rare skin condition that is a cutaneous manifestation of glucagonoma syndrome. It appears as an annular eruption of migrating erythematous papules and plaques with crusted erosions mostly in the intertriginous areas, central flaccid bullae, and superficial epidermal necrosis.^[11]^

It is found in as many as 90% of patients. It is widespread, primarily affecting the perioral, perigenital, and extremity regions. Most of the time, NME begins as red, itchy, and painful erythematous papules or plaques that eventually grow to become bullous lesions. Additionally, patients may have nail dystrophy and hair loss. One of the most common symptoms of glucagonoma syndrome is weight loss, which occurs in about 90% of patients.^[12]^ Nearly eighty percent of patients have diabetes; however, as the pancreas maintains beta cell function and produces insulin-reducing ketoacidosis, it is mostly mild. Thrombosis of the deep veins is seen in nearly half of the patients. A thorough history, physical, and examination for the possibility of glucagonoma should be performed on patients with unexplained thromboembolic disease. It may occasionally present as a potentially fatal pulmonary embolism.^[12]^

Chronic dermatosis may be connected to depression, which affects about half of the patients. Dementia, psychosis, agitation, hyperreflexia, ataxia, paranoid delusions, and proximal muscle weakness are additional neuropsychiatric manifestations. About 30% of patients with glucagonoma syndrome have persistent diarrhea.^[12]^

### 3.4. Vipoma

An uncommon paraneoplastic condition known as watery diarrhea, hypokalemia, and achlorhydria (WDHA) is brought on by an excess of vasoactive intestinal polypeptide (VIP) secreted by certain tumors. The syndrome begins slowly, and diagnosis typically takes months to years. Long-term dehydration and electrolyte and acid-base disturbances that cause chronic renal failure are linked to WDHA syndrome morbidity and mortality if they are not treated.^[13]^

Most of the time, patients with VIPoma have secretory, watery diarrhea that lasts even after a 48-hour fast. Before being diagnosed, diarrhea may have been present for several years. These odorless, tea-coloured stools result in significant fluid and electrolyte loss, including potassium.^[14]^

Due to dehydration and hypokalemia, additional symptoms include lethargy, nausea, vomiting, muscle weakness, and cramps. About 8% to 20% of patients have reported experiencing symptoms of vomiting. Hypomagnesemia is the cause of tetany, as has been documented. Heart arrhythmias, myopathy, tetany, and hypovolemic shock are all possible outcomes of severe fluid and electrolyte loss. In addition to persistent diarrhea, children with VIPoma may present with failure to thrive.^[14]^

### 3.5. Somatostatinoma

Somatostatinomas that are asymptomatic are usually nonfunctional. As the disease progresses, they may present with abdominal pain, vomiting, jaundice, and steatorrhea. This is due to the blockage of the biliary and pancreatic drainage by the tumor.^[15,19]^ Somatostatin inhibitory effects on the endocrine system are evident in functional tumors. They present with cholelithiasis in almost 70% of symptomatic cases and diabetes mellitus in 60% of symptomatic cases.^[16]^ In rare cases, somatostatinoma can present as a triad of diabetes mellitus, cholelithiasis, and steatorrhea. Due to the suppression of insulin, cholecystokinin, and pancreatic exocrine enzymes, this is known as the inhibitory syndrome.^[17,18]^ When a patient has obstructive jaundice, a physical examination may reveal icterus, abdominal discomfort indicating acute cholecystitis, and neurocutaneous indicators like cafe au lait spots, neurofibromas, and axillary freckling indicating a patient has concurrent neurofibromatosis 1 (NF1).^[19]^ Table 1 summarizes the clinical characteristics of F-PanNETs and their associated genetic syndromes.

**Table 1:** Summary of the clinical characteristics of functional pancreatic neuroendocrine tumors and their associated genetic syndromes.

Type of functional pancreatic neuroendocrine tumor	Hormone secreted	Prevalence (cases per population)	Common symptoms	Associated genetic syndromes
Insulinoma^[4–6,20–22]^	Insulin	1–4 cases per 1000,000	Hypoglycemic symptoms (change in behavior, confusion, seizures) diaphoresis, hunger, tremors, palpitations	MEN1, NF1
Gastrinoma^[8,9,20–22]^	Gastrin	0.5–3 per 1000,000	Abdominal pain, persistent diarrhea, dyspepsia	MEN1, NF1
Glucagonoma^[10–12,22]^	Glucagon	0.01–0.1 new cases per 100,000	Necrolytic migratory erythema (NME), painful glossitis, cheilitis, stomatitis, weight loss, diabetes mellitus, thromboembolic disease	MEN1
Somatostatinoma^[15,19,22]^	Somatostatin	1 in 40,000,000	abdominal pain, vomiting, jaundice, and steatorrhea	NF1
VIPOMA^[13,14,20]^	Vasoactive intestinal peptide (VIP)	1 in 1000,000	Watery diarrhea, hypokalemic symptoms (muscle weakness and cramps)	MEN1

MEN1 = multiple endocrine neoplasia 1, NF1 = neurofibromatosis 1, NME = necrolytic migratory erythema, VHL = Von Hippel-Lindau, VIP = vasoactive intestinal peptide.

## 4. Cancer-predisposition syndromes

Although the majority of panNETs are sporadic, up to 10% may arise in the context of familial syndromes due to inherited germline mutations in tumor suppressor genes (TSG).^[20,21]^ These syndromes are multiple endocrine neoplasia type 1 (MEN1), Von Hippel-Lindau disease (VHL), neurofibromatosis type 1 (NF1), and tuberous sclerosis complex (TSC), and are characterized by mutations in tumor suppressor genes (TSGs) in MEN1, VHL, NF1, and TSC1 or TSC2, respectively.^[20]^

### 4.1. Multiple endocrine neoplasia type 1

MEN1 is the inherited syndrome that is most frequently associated with the development of PanNETs.^[22]^ It is an autosomal dominant clinical syndrome that is present in approximately 3 per 100,000 individuals and has a penetrance of over 90% by age 40.^[23]^ Individuals with MEN1 frequently present with tumors of the parathyroid gland (95%), the anterior pituitary (20%–40%), and tumors originating from endocrine cells of the pancreas and duodenum (40%–80%).^[20–23]^

MEN1 syndrome arises in the context of an inactivating mutation of a tumor suppressor gene (TSG) located on chromosome 11q13.^[22,23]^ The MEN1 gene encodes the corresponding protein, named menin, which is ubiquitously expressed at the nuclear level.^[23]^ Menin is thought to be a scaffold protein involved in a wide variety of biological functions, including chromatin modification, inactivation of transcription factors, modulation of cell cycle inhibitors, and involvement with DNA repair mechanisms.^[22,23]^ Of note, MEN1 has been identified as the most frequently mutated gene in sporadic PanNET cases with no apparent familial history.^[21]^ Over 1300 mutations in MEN1 have been described to date, with more than 40% of sporadic cases displaying abnormally low nuclear staining of menin.^[24]^ In both inherited and sporadic cases, MEN1 mutations in pancreatic B cells result in the development of islet cell hyperplasia or dysplasia.^[24]^ The high rate of MEN1 mutations observed in both sporadic and syndromic PanNETs provides strong evidence that mutation of MEN1 plays a crucial role in pathogenesis.^[22,24]^

MEN1 syndrome PanNET cases and sporadic cases are similar in a clinical sense; however, MEN1 syndrome cases tend to have a younger age at presentation.^[22]^ 80% of MEN1 syndrome-related PanNETs are nonfunctioning PanNETs (NF-PanNETs), 54% are gastrinomas, 15-20% are insulinomas, 3% are glucagonomas, and rarer forms include VIPomas and GRFomas.^[22]^ Of all neoplasms that arise in the context of MEN1 syndrome, NF-PanNETs are most frequently associated with patient mortality, with 10-year disease-specific survival rates of 23% to 62%.^[20]^

Rarely, PanNETs may develop in association with multiple endocrine neoplasia type 4 (MEN4) due to CDKN1B germline mutations.^[21]^ CDKN1B encodes the cyclin-dependent kinase inhibitor p27, with mutations resulting in increased cell cycle progression and ultimately proliferation.^[21]^ Further studies are required to determine the penetrance of CDK1B mutations.^[23]^

### 4.2. Von Hippel-Lindau disease

Von Hippel-Lindau disease (VHL) is an autosomal dominant condition that arises as a result of germline mutations in the VHL TSG on chromosome 3p25–26,^[22]^ and has an incidence of around 1 in 36,000 individuals.^[23]^ VHL is characterized by the presence of retinal or cerebellar hemangioblastomas, pheochromocytomas, paragangliomas, renal clear cell carcinomas, and pancreatic cysts, or PanNETs.^[22,23]^

The VHL gene encodes 2 proteins that are involved in assembling the ubiquitin complex, which binds and inactivates hypoxia-inducible factor (HIF) 1a in normoxic conditions.^[22]^ Mutations in the VHL proteins result in a lack of degradation of HIF and resultant promotion of angiogenesis and tumor growth through increased transcription of hypoxia-response genes (e.g., vascular endothelial growth factor [VEGF]).^[20]^ This process ultimately leads to vascularized and cystic tumors.^[21]^

Patients with VHL syndrome often develop pancreatic serous cystadenomas, PanNETs, and mixed serous cystadenoma-PanNETs.^[20]^ PanNETs are seen in 5% to 17% of VHL patients, with the majority (80–100%) being NF-PanNETs, which are small and well-differentiated but can be locally aggressive and even metastatic.^[20,22]^ VHL-associated PanNETs have a better prognosis than sporadic cases, and VHL mutations rarely occur in association with sporadic cases.^[22]^ There have been rare reports of pancreatic somatostatinomas or insulinomas in association with VHL.^[21]^

### 4.3. Neurofibromatosis type 1

NF1 is an autosomal dominant condition that arises as a result of germline mutations in TSG NF1 on chromosome 17q11.^[20,22]^ NF1 has an incidence of 1 in 3000 and a very high penetrance, characterized by café-au-lait skin lesions, neurofibromas, and endocrinopathies.^[22,23]^

NF1 encodes the protein neurofibromin, which plays an important role in the downregulation of the mitogen-activated protein kinase and in the inhibition of the phosphatidylinositol 3-kinase (PI3K) pathway, which is involved in cell growth and proliferation.^[20,21]^

Rare cases of gastrinomas, insulinomas, and NF-PanNETs have been reported in association with NF1.^[20,21]^

### 4.4. Tuberous sclerosis complex

Tuberous Sclerosis Complex (TSC) is an autosomal dominant syndrome that arises as a result of mutations in TSC1 and TSC2 on chromosomes 9q34 and 16p13, respectively.^[23]^ TSC has an incidence of 1 in 10,000 and presents with hypomelanotic macules, angiofibromas, hamartomas, and neurological disorders.^[22,23]^

**Figure 1.**
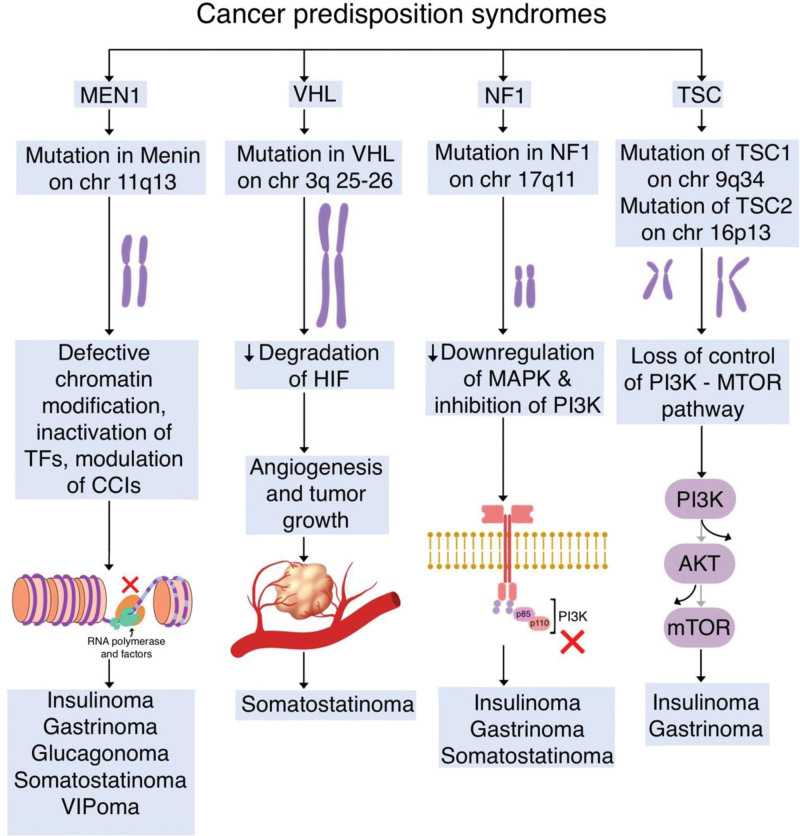
Illustration of the cancer predisposition syndromes associated with functional pancreatic neuroendocrine tumors. CCIs = cell cycle inhibitors, Chr = chromosome, HIF = hypoxia inducible factor, MAPK = mitogen activated protein kinase, MEN1 = multiple endocrine neoplasia 1, MTOR = mammalian target of rapamycin, NF1 = neurofibromatosis 1, PI3K = phosphatidylinositol-3-kinase, TFs = transcription factors, TSC = tuberous sclerosis complex, VHL = Von Hippel Lindau.

TSC1 and TSC2 proteins control cell proliferation through their roles in PI3K-mTOR pathway activity and insulin receptor signaling.^[22]^ The development of panNETs in the context of TSC is less frequent than in the other familial syndromes, ranging from 1.8% to 9%, with the majority being NF-PanNETs.^[20,22]^ TSC2 mutations are also found in approximately 8% of sporadic cases.^[22]^ In rare cases of TSC, patients develop functional panNETs, primarily insulinomas and gastrinomas.^[25]^ Figure 1 displays the cancer predisposition syndromes associated with the development of F-PanNETs.

## 5. Genetic landscape of PanNETs

### 5.1. Genetic landscape of sporadic PanNETs

The most common genetic event seen in sporadic PanNETs are MEN1 somatic mutations, which are present in 40 to 56% of cases.^[25]^ Notably, multiple loss of heterozygosity (LOH) analyses have reported allelic loss of MEN1 in up to 70% of sporadic PanNETs, and MEN1 mutations are usually associated with deletion of the other functional allele, resulting in complete loss of menin activity.^[25,26]^

DAXX and ATRX are implicated in chromatin stabilization, specifically in relation to telomeric chromatin.^[24]^ Loss of DAXX/ATRX is associated with alternative lengthening of telomeres (ALT), leading to unlimited cell cycling in neoplastic PanNET cells.^[27]^ 43% of sporadic PanNETs are associated with mutations in DAXX (25%) and ATRX (17.6%).^[21]^

The PI3K-Akt mTOR pathway is a “master cancer regulator” that is integral to many cellular processes, including angiogenesis, cell cycle progression, DNA repair, apoptosis, and epigenetic regulation.^[21]^ Mutations in genes encoding proteins involved in the pathway can be seen in almost 16% of well-differentiated sporadic PanNETs.^[20]^ The most commonly mutated PI3K-Akt mTOR pathway genes include PTEN (7%) and TSC2 (4%).^[28]^ The expression and activity of mTOR have been seen to be higher in PanNET tissue than in normal pancreatic tissue.^[29]^

In one study of the genetic alterations underpinning sporadic PanNET pathogenesis, 63% of all cases were seen to have had one or more mutations in MEN1/DAXX/ATRX.^[20]^

### 5.2. Genetic landscape of insulinomas

While there are pathological similarities between insulinomas and NF-PanNETs, it remains controversial as to whether there is a common genetic basis with regards to somatic mutations. MEN1, DAXX/ATRX, and mTOR pathway genes are frequently mutated in patients with NF-PanNETs; however, these mutations are less frequently observed in insulinomas.^[30]^ In contrast, recurrent YY1 mutations are seen in 15 to 32% of insulinomas, but similar mutations are not seen in NF-PanNETs.^[31]^

The Yin Yang 1 (YY1) gene is a transcriptional activator of the INS gene, which plays a role in Ca2+-dependent insulin secretion.^[32]^ YY1 mutations have been observed to be driver mutations in insulinomas^[31,33]^; however, no other significantly mutated genes have been identified. YY1 mutations are of little prognostic value, as similar proportions were found in both indolent and aggressive insulinomas.^[30]^ One study of the mutational profiles and copy number variation (CNV) patterns of 84 insulinomas found that Y11-mutated insulinomas tended to belong to the copy-neutral subtype, whereas insulinomas with no Y11 mutations had a high prevalence of CNV amplification.^[31]^

Loss of chromosome 6q and gains of 12q, 14q, and 17pq have been associated with aggressive insulinomas, and chromosomal instability (CIN) was the best predictor of aggression in a series of 62 insulinomas.^[30]^ Alternative lengthening of telomeres (ALT) is a possible cause of CIN, and in 1 study, it was seen in 4/5 insulinomas while absent in 30 indolent cases.^[34]^ Aggressive insulinomas regularly harbor mutations of ATRX and DAXX that are virtually non-existent in indolent insulinomas, supporting the theory that aggressive insulinomas may originate from NF-PanNETs. Several cases of NF-PanNETs progressing to aggressive insulinomas have been published.^[35–37]^

### 5.3. Genetic landscape of gastrinomas

Gastrinomas are associated with MEN1 syndrome in 25 to 54% of patients.^[22,38]^ Sporadic gastrinomas are characterized by high rates of MEN1 somatic mutations, between 31 and 58%.^[38]^ Interestingly, rates of copy number variations are very low in gastrinomas, in contrast with the higher rates seen in NF-PanNETs and insulinomas.^[38]^ In 1 study of 27 gastrinomas, deletions in 1q were detected in 44% and were associated with aggressive growth and liver metastases.^[39]^ In another study, a loss of 3p was observed in 19% and a gain of 9p was observed in 29% of sporadic gastrinomas. Losses at 11q13 were also a relatively frequent event, occurring in about 15-20% of gastrinomas.^[22]^ The gain of 17q is frequently seen in insulinomas and may implicate HER2/NEU, which is located on chromosome 17q21. HER2/NEU gene amplifications are a negative prognostic marker in breast and gastroesophageal tumors and have been identified in 45% of gastrinomas in 1 study.^[40]^ Missiaglia et al observed LOH of the X chromosome in 40% of female patients and loss of the Y chromosome in 36% of male patients. These sex chromosome alterations were associated with metastasis and a worse prognosis.^[41]^ Finally, the tumor suppressor gene CDKN2A is inactivated more frequently in gastrinomas than in either NF-PanNETs or insulinomas.^[22]^

### 5.4. Genetic landscape of glucagonomas, VIPomas and somatostatinomas

The genetic mechanisms involved in the malignant progression of glucagonomas are still largely unknown. 3% of those with MEN1 syndrome develop glucagonomas,^[25,42]^ and mutations in MEN1 have been observed in approximately 67% of sporadic glucagonomas,^[43,44]^ compared to just 7% in insulinomas. LOH of 11q13 has also been observed in advanced glucagonomas.^[25]^ In 1 study of 4 glucagonomas, there were a high number of genetic aberrations per tumor (mean 12.8), with 7q gain present in all.^[45]^

VIPomas are rarely associated with MEN1 syndrome; however, somatic mutations in MEN1 have been found in 44% of sporadic VIPomas.^[22]^

Somatostatinomas are associated with MEN1 syndrome in 40 to 50% of familial cases and also with NF1 and VHL syndromes. There are a variety of chromosomal aberrations associated with sporadic somatostatinomas, including LOH at chromosomes 11q and 6q and allelic loss at 3p.^[19]^ Somatostatinomas may be associated with tumor-specific mutations of hypoxia-inducible factor 2a, which is encoded by EPAS1, which may result in the syndrome of paraganglioma and somatostatinoma associated with polycythemia.^[23,46]^

## 6. Histopathology, immunohistochemistry and biomarkers

Several studies have investigated the use of biomarkers in PanNETs and shown promising results.^[47]^ However, most studies have discussed the biomarkers of gastroenteropancreatic neuroendocrine tumors, and fewer have focused on the biomarkers of F-PanNETs. Regarding PanNETs, one of the most widely reported biomarkers is chromogranin A (CgA). CgA is a protein that is secreted by the neuroendocrine cells of the pancreas and is often elevated in patients with PanNETs, being considered a good diagnostic and prognostic biomarker.^[48–50]^ Additionally, synaptophysin is a protein that is expressed in the neuroendocrine cells of the pancreas and is often used as an immunohistochemical marker for PanNETs, as well as a useful prognostic marker,^[51–53]^ as it is associated with worse patient outcomes.^[53]^

Insulinomas pose significant diagnostic challenges due to their small size and diverse clinical presentations. In recent years, advancements in understanding the molecular underpinnings of insulinomas have shed light on potential biomarkers that can aid in the early detection and accurate classification of these tumors.^[54]^ Novel biomarkers, including microRNA signatures and circulating neuroendocrine markers, have emerged as promising noninvasive tools to complement traditional diagnostic methods. Indeed, glucose, insulin, and C-peptides have all emerged as reliable indicators.^[55]^ It should be noted that insulinomas tend to remain smaller than F-PanNETs, making the diagnosis more difficult. Such parameters are, therefore, key to the diagnostic process.^[54,55]^ Still, CgA has been shown to be a useful biomarker in PanNETs, but not as much in the case of insulinomas.^[56]^

One of the primary biomarkers utilized in the diagnosis of gastrinomas is the measurement of circulating gastrin levels, which often display marked elevation in affected individuals.^[57]^ Nevertheless, due to the overlap in gastrin levels with other gastrointestinal disorders, this biomarker alone may not always be sufficient for a definitive diagnosis.^[58]^ Contrasting data exists on CgA, which, while it has emerged as an option and is a sensitive marker for assessing overall neuroendocrine activity, seems to be less specific than gastrin or progastrin.^[57]^ Furthermore, the evaluation of expression patterns of somatostatin receptors, particularly SST2A, through immunohistochemical techniques has garnered attention as a potential biomarker for identifying patients who may benefit from targeted peptide receptor radionuclide therapy.^[59]^

One of the most significant biomarkers utilized in the assessment of VIPomas is VIP itself, which is responsible for the characteristic clinical syndrome of WDHA.^[60]^ Elevated circulating levels of VIP serve as a diagnostic hallmark, though caution must be exercised as other non-VIPoma-related conditions may also lead to elevated VIP levels.^[61]^ In light of this, combinatorial biomarker strategies have been investigated, involving the assessment of CgA and neuron-specific enolase (NSE), that may enhance diagnostic accuracy.^[62]^

Glucagon, the hormone secreted by glucagonomas, serves as a crucial biomarker for diagnosing glucagonoma syndrome, characterized by diabetes mellitus, dermatitis, glossitis, and weight loss.^[63]^ Elevated circulating glucagon levels, in conjunction with other clinical and imaging findings, help to establish the diagnosis.^[63]^ However, given the overlap of glucagon levels with other pancreatic disorders, a complementary approach using additional biomarkers, such as CgA and pancreatic polypeptide, has been explored to enhance diagnostic accuracy.^[63]^

The key biomarker utilized in the diagnostic evaluation of somatostatinomas is somatostatin itself, whose excessive secretion contributes to the characteristic somatostatinoma syndrome, comprising diabetes mellitus, cholelithiasis, diarrhea, and weight loss.^[64]^ Elevated circulating levels of somatostatin aid in establishing the diagnosis, although the overlap of somatostatin levels with other pancreatic disorders necessitates the integration of additional biomarkers for enhanced diagnostic precision.^[64]^

Histopathology and immunohistochemistry tests play a crucial role in the diagnosis of F- and PanNETs.^[65]^ Immunohistochemistry has become a useful ancillary study in the identification and classification of pancreatic neoplasms. Diagnostic accuracy has been significantly improved because of the continuous discoveries of tumor-associated biomarkers and the development of effective immunohistochemical panels.^[66]^ The application of appropriate immunohistochemical panels enables pathologists to differentiate pancreaticobiliary adenocarcinomas from reactive conditions and to identify and classify pancreatic neoplasms. Tested markers may include glucose or insulin (for insulinoma), gastrin or gastric pH (for gastrinoma), and glucose or glucagon (for glucagonoma). Apart from the aforementioned biochemical markers, further markers are utilized in immunohistochemical analysis. The Ki-67 proliferation index can be used as an addition to histologic grading and is considered to be an indicator for disease recurrence and prognosis.^[65,67]^ In addition, several other markers, including insulin-associated protein 1^[68]^ and Phosphorylated histone H3, are associated with the worst prognosis and early progression.^[69]^

## 7. Investigations and diagnosis

Due to the secretion of various hormones amongst the F-PanNETs, distinct clinical features are present; however, considerable clinical expertise is required to recognize hormonal hypersecretion symptoms. In the case of an insulinoma, the new onset of hypoglycemic symptoms in a non-diabetic patient should raise suspicion of an underlying cause. This is also true of gastrinomas, somatostatinomas, etc. Clinical suspicion of a potential functional neuroendocrine tumor that arises due to the presence of hormonal hypersecretion symptoms should prompt further investigations. Somatostatinoma presents with extremely vague symptoms, and thus its history may not be as remarkable compared to the other subtypes of F-PanNETs. Physical examination is often unremarkable unless there has been metastasis, with the most common site being the liver, which may present with jaundice and hepatomegaly.^[70]^

Blood and serum testing to assess hormone levels should be performed promptly following a history and examination. Fasting serum levels of insulin, proinsulin, glucagon, VIP, and gastrin may be helpful in identifying excess levels of these hormones, which may aid in the diagnosis of a F-PanNET.^[2]^ Blood tests for biomarkers such as CGA, as mentioned previously, may also be utilized; however, they are commonly associated with false-positive results, especially in those taking anti-acid medication or having atrophic gastritis.^[71]^

Imaging plays a huge role in the diagnosis of F-PanNETs. A number of modalities have been used in the diagnostic approach: computed tomography (CT), magnetic resonance imaging (MRI), positron emission tomography scan, and ultrasound.^[72]^ CT has a sensitivity of 89 to 97% for pancreatic cancer, which is equivalent to MRI.^[73]^ A positron emission tomography scan is well known for its ability to detect metastasis and is thus a useful tool in the assessment of advanced disease. Ultrasound, specifically endoscopic ultrasound (EUS), has the unique advantage, as compared to other imaging modalities, of allowing for direct visualization of pancreatic lesions in real time and allowing for a biopsy to be obtained at the time of assessment via fine-needle aspiration, which is required to provide a tissue sample for definitive diagnosis with histopathological analysis.

According to a recent consensus of guidelines for the management of F-PanNETs,^[74]^ it is stated that somatostatin receptor scintigraphy is useful in the staging of these tumors, and imaging with positron emission tomography with CT with gadolinium-68 labeled somatostatin analogues has the highest sensitivity for localization (86–100%) and specificity of 79–100% for all PanNETs except in the case of insulinomas with a sensitivity of only 25%, thus requiring more advanced modalities. One such imaging technique that is positive in 90–100% of cases is intra-arterial injection of calcium with hepatic venous insulin gradients (IACIG), and this study recommends IACIG be considered when other imaging tests are negative. A biopsy with subsequent histopathological analysis is the gold standard of diagnosis and should be sought in every situation. EUS allows for both assessment of the lesion and biopsy, as mentioned previously; however, CT, MRI, or US guided procedures may also be used to acquire a tissue sample from sites of metastasis, such as the liver.^[71]^

## 8. Current management guidelines and advancements in targeted therapies

The American Society of Clinical Oncology (ASCO) provides guidelines for the management of pancreatic cancer in the United States. These guidelines recommend multidisciplinary care for all patients with pancreatic cancer, including those with PanNETs. They recommend surgical resection for localized tumors and medical therapy with somatostatin analogues (SSAs), Everolimus, or sunitinib for patients with unresectable or metastatic PanNETs.^[75]^ The European Society for Medical Oncology (ESMO) provides guidelines for the management of neuroendocrine neoplasms (NENs) in Europe. These guidelines recommend surveillance for small, asymptomatic PanNETs <2 cm in size and surgical resection (enucleation, pancreatectomy) for localized PanNETs. The guidelines also recommend the use of SSAs for patients with functional tumors and targeted therapy for patients with nonfunctional tumors or progressive disease.^[76]^

The National Institute for Health and Care Excellence (NICE) recommends referral to a specialist center for all patients with suspected pancreatic cancer, including PanNETs. The guidelines recommend surgical resection for localized PanNETs and medical therapy with SSAs or targeted therapy with Everolimus or sunitinib for patients with unresectable or metastatic PanNETs.^[77]^ The Japanese Society for Cancer of the Colon and Rectum (JSCCR) also recommends resection for localized PanNETs and medical therapy with SSAs or targeted therapy with Everolimus or sunitinib for patients with unresectable or metastatic PanNETs.^[78]^ The Chinese Society of Clinical Oncology (CSCO) recommends surgical resection for localized PanNETs and medical therapy with SSAs, Everolimus, or sunitinib for patients with unresectable or metastatic PanNETs. They also recommend regular monitoring with imaging, biomarkers, and histopathology. In addition, the guidelines provide specific recommendations for the management of functional and nonfunctional PanNETs, taking into account the unique characteristics of the Chinese population, such as a higher prevalence of nonfunctional PanNETs and a lower incidence of MEN1.^[79]^

A new guidance paper published by the European Neuroendocrine Tumor Society (ENETS) in May 2023 has set out several important recommendations in regard to the management of F-PanNETs.^[80]^ In regard to insulinomas, parenchyma-sparing pancreatic resections such as enucleation and central pancreatectomy should be considered as a first-line option using minimally invasive techniques if feasible. EUS radiofrequency ablation (EUS-RFA) may be considered for patients with < 2cm localized insulinomas who are deemed unfit for surgery. Resection of the primary tumor and systematic lymphadenectomy should be the treatment of choice for sporadic gastrinoma without disseminated disease. Those with MEN1-ZES should be counseled with regard to the risks and benefits of undergoing a pancreaticoduodenectomy due to the higher chance of cure with this procedure. Patients with advanced disease require a multi-modal approach involving supportive, surgical, interventional, hormonal, and anti-proliferative therapies; cytoreductive surgery may be considered for patients when > 70 to 90% of visible disease can be resected. Other locoregional therapies include EUS-RFA or chemotherapy or radioembolization. When supportive therapy fails for advanced disease, palliative SSA therapy may be considered. This paper states that peptide receptor radionuclide therapy, chemotherapy, and Everolimus or sunitinib should be reserved for SSA-resistant individuals patients with advanced disease should subsequently be followed up according to the grade of tumor, bulk, growth rate, and primary treatment modality, and thus an individualized approach is recommended.

The emergence of targeted therapies has led to a paradigm shift in the treatment of panNETs. One is the mTOR pathway, which plays a critical role in cell growth and proliferation. Everolimus is a targeted therapy that inhibits this pathway and has been shown to improve survival in patients with advanced disease.^[81]^ Indeed, the RADIANT-3 trial showed a significant improvement in patients who received Everolimus compared to placebo, leading to its approval by the FDA for the treatment of unresectable and metastatic panNETs.^[83]^ However, Everolimus has also been associated with adverse effects, including fatigue, mucositis, and hyperglycemia, which need to be monitored.^[81,82]^ Another pathway that has been targeted in PanNETs is the VEGF pathway, which plays a critical role in angiogenesis and tumor growth.^[83]^ Sunitinib inhibits the VEGF pathway and has been shown to improve survival in patients with advanced disease.^[84]^ The SUN-1111 trial demonstrated a significant improvement in survival with sunitinib compared to placebo, especially in patients with unresectable and metastatic disease.^[85]^

Despite the recently developed targeted therapies, there are still challenges that need to be addressed. One challenge is the identification of additional relevant targets beyond the mTOR and VEGF pathways to provide support and options for more patient groups with fewer side effects due to treatments. Recent studies have identified potential targets such as the insulin-like growth factor-1 receptor (IGF-1R), which may be a further promising target for PanNETs management.^[86]^

**Figure 2.**
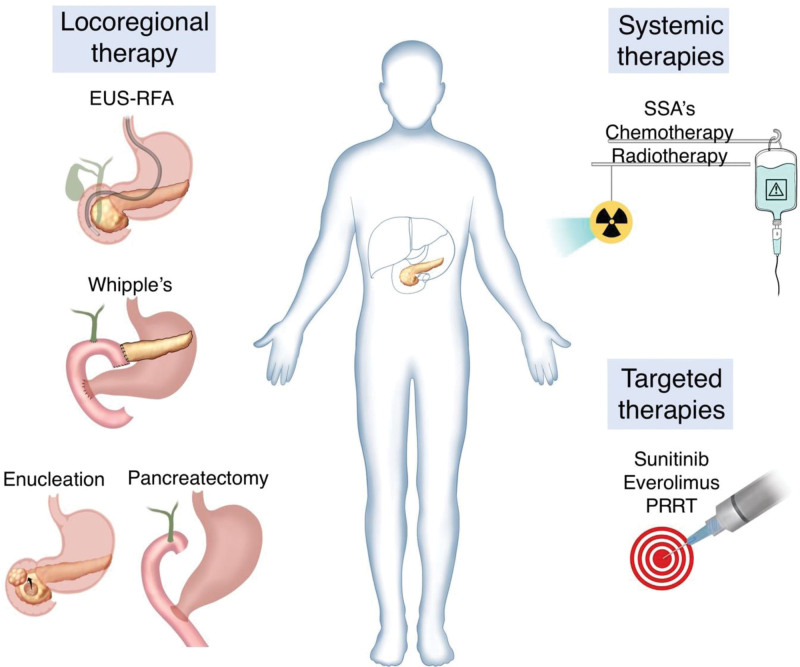
Summary of treatment options available in the management of functional pancreatic neuroendocrine tumors EUS-RFA = endoscopic ultrasound—radiofrequency ablation, PRRT = peptide receptor radionuclide therapy, SSA = somatostatin analogues.

Another challenge is the development of resistance to therapies. Combination therapies, such as the combination of everolimus and sunitinib, may be effective in overcoming resistance and improving outcomes in patients with different tumor types.^[87]^ The RADIANT-4 trial demonstrated a significant improvement in progression-free survival with the combination of everolimus and sunitinib compared to everolimus alone in PanNETs patients.^[88]^ Figure 2 presents a summary of validated treatment options used in the management of F-PanNETs.

## 9. Prognosis

Larger tumor size (> 2 cm), regional or distant disease, and the presence of gastrinomas or NF-PanNETs are predictors of death in the context of F-PanNETs. These factors can be useful in clinical decision-making and risk assessment. Additionally, surgery plays a crucial role in increasing the survival time of patients with F-PanNETs, as it has been shown to improve disease-related surgery is the standard treatment for F-PanNETs, and it generally yields a good prognosis. However, if the disease is locally advanced or has metastasized, the mortality rate increases significantly, and the available treatment options have limited effectiveness.^[89,90]^

These findings align with previous research indicating that F-PanNETs generally have a more favorable prognosis compared to NF-PanNETs and exhibit better treatment outcomes.^[90,91]^

Tumors larger than 2 cm were independently associated with an elevated risk of death. This observation is consistent with multiple studies that have demonstrated how insulinomas larger than 2 cm are predictive of metastatic disease, and gastrinomas larger than 3 cm indicate a poorer prognosis. These findings suggest that a larger tumor size (> 2 cm) influences the biological behavior of F-PanNETs, leading to worse outcomes, while smaller tumors are associated with a more favorable prognosis.^[90,92,93]^

Recent advancements in surgical treatment, interventional radiology, ablation techniques, and targeted therapies have contributed to improved treatment outcomes and, consequently, better prognosis for F-PanNET patients. Several factors independently correlate with an elevated risk of survival.^[90,94–99]^

### 9.1. Insulinoma

Recent research has shed light on valuable prognostic factors for insulinoma, aiding in the prediction of metastatic disease and tumor-free survival. Specific chromosomal alterations, such as 3p and 6q loss, as well as 7q, 12q, and 14q gain, have been identified as strong indicators of tumor recurrence, progression, or tumor-specific death in insulinomas. Furthermore, molecular markers like Ki67 and CK19 expression have shown significance in prognostication. Combining these molecular markers with tumor size and chromosomal instability (CIN) further enhances their predictive value. CIN, in particular, has emerged as the most reliable indicator of metastatic disease, with a sensitivity of 85%. When combined with tumor size or 6q loss, the sensitivity increases to 92%. Moreover, combining CIN with Ki67 expression achieves a sensitivity of 100%. However, the literature lacks clarity regarding survival differences between local and regional disease in insulinoma patients.^[92]^

Liver metastases play a pivotal role in the prognosis of patients with endocrine tumors of the digestive pancreas (ETDP). The progression of liver metastases and the complete resection of the primary tumor are important prognostic factors. Tumoral cell differentiation also influences prognosis in these cases.^[100]^

### 9.2. Glucagonoma

Despite most patients being diagnosed at an advanced stage, the prognosis for glucagonoma remains relatively good due to its slow tumor growth. Mean survival estimates after diagnosis have ranged from 3 to 7 years or even longer. It is noteworthy that the ultimate cause of death in many glucagonoma patients is unrelated to the tumor itself. Patients who succumb to tumor-related deaths often experience complications such as thromboembolism, sepsis, or gastrointestinal bleeding.^[101]^

### 9.3. Gastrinoma

A cure is a realistic possibility for a significant proportion (50%) of patients with sporadic gastrinoma. The introduction of duodenotomy, a surgical procedure involving the opening of the duodenum, has contributed to both improved tumor detection rates and increased cure rates. Routine duodenotomy has become an essential approach to managing gastrinoma cases by enabling the identification and removal of additional duodenal gastrinomas, thus improving patient outcomes.^[19,102]^

### 9.4. Somatostatinoma

The prognosis for pancreatic and periampullary somatostatinomas hinges on several factors. Localised disease is associated with a 5-year survival rate of 60–100%, while metastatic disease has a lower rate of 15 to 60%. Prognostic markers indicating a poor outcome include large tumor size (>3cm), poor differentiation, and lymph node involvement. nonfunctioning somatostatinomas, which are typically poorly differentiated, exhibit a worse prognosis compared to functioning somatostatinomas.^[103]^

### 9.5. VIPoma

Patients with VIPoma experience a median survival of 96 months. Prognosis depends primarily on tumor grade, stage, and the feasibility of surgical resection. The National Comprehensive Cancer Network (NCCN) guidelines recommend regular post-resection follow-up, including history and physical examination, multiphasic CT or MRI scans, and serum VIP level monitoring during the initial 3- to 12-month period. After the first year, these measures should be repeated every 6 to 12 months to ensure timely detection of any recurrence or progression of the disease.^[12]^

## 10. Conclusion and future directions

It is clear to see that F-PanNETs is an extremely interesting, rare group of neoplasms. Much work has been performed thus far in regards to the study of the molecular underpinnings of this condition, which has allowed for us to create novel therapies and improve outcomes for sufferers of this condition. We hope our study will consolidate the available literature on this topic and become a source of education for clinicians and researchers, as well as raise awareness for this rare condition.

In regard to future directions in this field, we believe greater research is required to identify novel biomarkers of F-PanNETs. As mentioned before, circulating levels of hormones appears to the most used method currently, however this approach requires further investigations in order to identify the cause of this observation. Tumor markers specific to F-PanNETs will aid in the diagnosis of this condition as well help minimize the use of invasive testing such EUS. We believe our study has exposed this lack of biomarkers in the literature. Moreover, further genetic studies will allow us to identify more potential molecular therapeutic targets, such as in the case with everolimus, which in turn may allow patients to avoid surgery and the associated risks.

## Author contributions

**Conceptualization:** Yasir Alshareefy.

**Data curation:** Yasir Alshareefy, Sinead Cummins, Adele Mazzoleni, Vidushi Sharma.

**Methodology:** Yasir Alshareefy, Sinead Cummins.

**Resources:** Amanda Weng Yee Leong.

**Supervision:** Andrew Wireko.

**Validation:** Andrew Wireko.

**Visualization:** Yasir Alshareefy, Saibaba Guggilapu, Amanda Weng Yee Leong.

**Writing – original draft:** Yasir Alshareefy, Sinead Cummins, Adele Mazzoleni, Vidushi Sharma.

**Writing – review & editing:** Yasir Alshareefy, Sinead Cummins, Adele Mazzoleni, Vidushi Sharma, Saibaba Guggilapu, Amanda Weng Yee Leong, Andrew Wireko.

## References

[1] Halfdanarson TR Rubin J Farnell MB. Pancreatic endocrine neoplasms: epidemiology and prognosis of pancreatic endocrine tumors. Endocr Relat Cancer. 2008;15:409–27. 18508996 10.1677/ERC-07-0221 PMC2693313

[2] Ro C Chai W Yu VE. Pancreatic neuroendocrine tumors: biology, diagnosis,and treatment. Chinese J Cancer. 2013;32:312–24. 10.5732/cjc.012.10295 PMC3845620 23237225

[3] Öberg K. Management of functional neuroendocrine tumors of the pancreas. Gland Surg. 2018;7:20–7. 29629316 10.21037/gs.2017.10.08 PMC5876681

[4] Placzkowski KA Vella A Thompson GB. Secular trends in the presentation and management of functioning insulinoma at the Mayo Clinic, 1987-2007. J Clin Endocrinol Metab. 2009;94:1069–73. 19141587 10.1210/jc.2008-2031

[5] StatPearls. Insulinoma. StatPearls. June 20, 2023. https://www.statpearls.com/articlelibrary/viewarticle/23569/ Accessed August 7, 2023.

[6] Hypoglycemia - StatPearls - NCBI Bookshelf. https://www.ncbi.nlm.nih.gov/books/NBK534841/ Accessed August 7, 2023.

[7] Karanth JB Pai V Maribashetti K. Pancreatic neuroendocrine tumour-insulinoma masquerading as a psychiatric illness. BMJ Case Rep. 2022;15:e249698. 10.1136/bcr-2022-249698 PMC9198684 35701018

[8] H; CSMG. Gastrinoma. National Center for Biotechnology Information. https://pubmed.ncbi.nlm.nih.gov/28722872/ Accessed August 7, 2023.

[9] Tonelli F Giudici F Nesi G. Biliary tree gastrinomas in multiple endocrine neoplasia type 1 syndrome. World J Gastroenterol. 2013;19:8312–20. 24363522 10.3748/wjg.v19.i45.8312 PMC3857454

[10] de Herder WW Hofland J. Glucagon & Glucagonoma Syndrome. In: Feingold KR Anawalt B Blackman MR, ., eds. Endotext. South Dartmouth (MA): MDText.com, Inc.; April 7, 2023.

[11] Tolliver S Graham J Kaffenberger BH. A review of cutaneous manifestations within glucagonoma syndrome: necrolytic migratory erythema. Int J Dermatol. 2018;57:642–5. 29450880 10.1111/ijd.13947

[12] Sandhu S Jialal I. GLUCAGONOMA syndrome - statpearls - NCBI bookshelf. Accessed August 7, 2023. https://www.ncbi.nlm.nih.gov/books/NBK519500/?report=reader.

[13] Grier JF. WDHA (watery diarrhea, hypokalemia, achlorhydria) syndrome: clinical features, diagnosis, and treatment. South Med J. 1995;88:22–4. 7817223 10.1097/00007611-199501000-00002

[14] Sandhu S Jialal I. Vipoma - StatPearls - NCBI Bookshelf - National Center for... Accessed August 7, 2023. https://www.ncbi.nlm.nih.gov/books/NBK507698/.

[15] Tanaka S Yamasaki S Matsushita H. Duodenal somatostatinoma: a case report and review of 31 cases with special reference to the relationship between tumor size and metastasis. Pathol Int. 2000;50:146–52. 10792774 10.1046/j.1440-1827.2000.01016.x

[16] Öberg K Knigge U Kwekkeboom D. ESMO Guidelines Working Group Neuroendocrine gastro-entero-pancreatic tumors: ESMO Clinical Practice Guidelines for diagnosis, treatment and follow-up. Ann Oncol. 2012;23(Suppl 7):viivii124–vii130. 10.1093/annonc/mds295 22997445

[17] Mozell E Stenzel P Woltering EA. Functional endocrine tumors of the pancreas: clinical presentation, diagnosis, and treatment. Curr Probl Surg. 1990;27:301–86. 1973365 10.1016/0011-3840(90)90025-z

[18] Nesi G Marcucci T Rubio CA. Somatostatinoma: clinico-pathological features of three cases and literature reviewed. J Gastroenterol Hepatol. 2008;23:521–6. 17645474 10.1111/j.1440-1746.2007.05053.x

[19] Elangovan A Zulfiqar H. Somatostatinoma - StatPearls - NCBI Bookshelf. Accessed August 7, 2023. https://www.ncbi.nlm.nih.gov/books/NBK551613/. 31869077

[20] Pea A Hruban RH Wood LD. Genetics of pancreatic neuroendocrine tumors: implications for the clinic. Expert Rev Gastroenterol Hepatol. 2015;9:1407–19. 26413978 10.1586/17474124.2015.1092383 PMC4890468

[21] Pipinikas CP Berner AM Sposito T. The evolving (epi)genetic landscape of pancreatic neuroendocrine tumours. Endocr Relat Cancer. 2019;26:R519–44. 31252410 10.1530/ERC-19-0175

[22] Capurso G Festa S Valente R. Molecular pathology and genetics of pancreatic endocrine tumours. J Mol Endocrinol. 2012;49:R37–50. 22586144 10.1530/JME-12-0069

[23] Crona J Skogseid B. GEP- NETS UPDATE: Genetics of neuroendocrine tumors. Eur J Endocrinol. 2016;174:R275–90. 27165966 10.1530/EJE-15-0972

[24] Zhang J Francois R Iyer R. Current understanding of the molecular biology of pancreatic neuroendocrine tumors. J Natl Cancer Inst. 2013;105:1005–17. 23840053 10.1093/jnci/djt135 PMC6281020

[25] Maharjan CK Ear PH Tran CG. Pancreatic neuroendocrine tumors: molecular mechanisms and therapeutic targets. Cancers (Basel). 2021;13:5117. 34680266 10.3390/cancers13205117 PMC8533967

[26] Chen M Van Ness M Guo Y. Molecular pathology of pancreatic neuroendocrine tumors. J Gastrointest Oncol. 2012;3:182–8. 22943010 10.3978/j.issn.2078-6891.2012.018 PMC3418536

[27] Fang JM Shi J. A Clinicopathologic and molecular update of pancreatic neuroendocrine neoplasms with a focus on the New World Health Organization Classification. Arch Pathol Lab Med. 2019;143:1317–26. 31509453 10.5858/arpa.2019-0338-RA PMC7141760

[28] Gao HL Wang WQ Yu XJ. Molecular drivers and cells of origin in pancreatic ductal adenocarcinoma and pancreatic neuroendocrine carcinoma. Exp Hematol Oncol. 2020;9:28. 33101770 10.1186/s40164-020-00184-0 PMC7579802

[29] Capurso G Archibugi L Delle Fave G. Molecular pathogenesis and targeted therapy of sporadic pancreatic neuroendocrine tumors. J Hepatobiliary Pancreat Sci. 2015;22:594–601. 25619712 10.1002/jhbp.210

[30] Hackeng WM Brosens LAA Dreijerink KMA. Aggressive versus indolent insulinomas: new clinicopathological insights. Endocr Relat Cancer. 2023;30:e220321. 36779771 10.1530/ERC-22-0321

[31] Hong X Qiao S Li F. Whole-genome sequencing reveals distinct genetic bases for insulinomas and non-functional pancreatic neuroendocrine tumours: leading to a new classification system. Gut. 2020;69:877–87. 31462556 10.1136/gutjnl-2018-317233 PMC7229893

[32] Liu D Yang KY Chan VW. YY1 regulates glucose homeostasis through controlling insulin transcription in pancreatic β-cells. Diabetes. 2022;71:961–77. 35113157 10.2337/db21-0695 PMC9044128

[33] Cao Y Gao Z Li L. Whole exome sequencing of insulinoma reveals recurrent T372R mutations in YY1. Nat Commun. 2013;4:2810. 24326773 10.1038/ncomms3810

[34] Hackeng WM Schelhaas W Morsink FHM. Alternative lengthening of telomeres and differential expression of endocrine transcription factors distinguish metastatic and non-metastatic insulinomas. Endocr Pathol. 2020;31:108–18. 32103422 10.1007/s12022-020-09611-8 PMC7250793

[35] Juhlin CC Skoglund S Juntti-Berggren L. Non-functioning neuroendocrine pancreatic tumors transforming to malignant insulinomas - four cases and review of the literature. Neuro Endocrinol Lett. 2019;40:175–83. 32087093

[36] Keen F Iqbal F Owen P. Metastatic insulinoma presenting 14 years after benign tumour resection: a rare case and management dilemma [published online ahead of print, 2020 Sep 23]. Endocrinol Diabetes Metab Case Rep. 2020;2020:20–0065. 10.1530/EDM-20-0065 PMC7576662 33434180

[37] Yu R. Malignant Insulinoma is largely derived from nonfunctioning pancreatic neuroendocrine tumors: a contemporary view. Pancreas. 2020;49:733–6. 32590616 10.1097/MPA.0000000000001562

[38] Tirosh A Kebebew E. Genetic and epigenetic alterations in pancreatic neuroendocrine tumors. J Gastrointest Oncol. 2020;11:567–77. 32655936 10.21037/jgo.2020.03.11 PMC7340809

[39] Chen YJ Vortmeyer A Zhuang Z. Loss of heterozygosity of chromosome 1q in gastrinomas: occurrence and prognostic significance. Cancer Res. 2003;63:817–23. 12591732

[40] Evers BM Rady PL Sandoval K. Gastrinomas demonstrate amplification of the HER-2/neu proto-oncogene. Ann Surg. 1994;219:596–601; discussion 602. 7911296 10.1097/00000658-199406000-00002 PMC1243202

[41] Missiaglia E Moore PS Williamson J. Sex chromosome anomalies in pancreatic endocrine tumors. Int J Cancer. 2002;98:532–8. 11920612 10.1002/ijc.10223

[42] Solcia E Klöppel G Sobin LH. Histological classification of endocrine tumours. Histological Typing Endocr Tumours. 2000:7–13.

[43] Moore PS Missiaglia E Antonello D. Role of disease-causing genes in sporadic pancreatic endocrine tumors: MEN1 and VHL. Genes Chromosomes Cancer. 2001;32:177–81. 11550286 10.1002/gcc.1180

[44] Di Domenico A Wiedmer T Marinoni I. Genetic and epigenetic drivers of neuroendocrine tumours (NET). Endocr Relat Cancer. 2017;24:R315–34. 28710117 10.1530/ERC-17-0012

[45] Speel EJ Richter J Moch H. Genetic differences in endocrine pancreatic tumor subtypes detected by comparative genomic hybridization. Am J Pathol. 1999;155:1787–94. 10595906 10.1016/S0002-9440(10)65495-8 PMC1866934

[46] Yang C Hong CS Prchal JT. Somatic mosaicism of EPAS1 mutations in the syndrome of paraganglioma and somatostatinoma associated with polycythemia. Hum Genome Var. 2015;2:15053. 27081557 10.1038/hgv.2015.53 PMC4785578

[47] Rossi RE Garcia-Hernandez J Meyer T. Chromogranin A as a predictor of radiological disease progression in neuroendocrine tumours. Ann Transl Med. 2015;3:118. 26207246 10.3978/j.issn.2305-5839.2015.04.23 PMC4481369

[48] Qiao XW Qiu L Chen YJ. Chromogranin A is a reliable serum diagnostic biomarker for pancreatic neuroendocrine tumors but not for insulinomas. BMC Endocr Disord. 2014;14:64. 25099181 10.1186/1472-6823-14-64 PMC4130880

[49] Choi JH Paik WH. Risk stratification of pancreatic neuroendocrine neoplasms based on clinical, pathological, and molecular characteristics. J Clin Med. 2022;11:7456. 36556070 10.3390/jcm11247456 PMC9786745

[50] Pulvirenti A Rao D Mcintyre CA. Limited role of Chromogranin A as clinical biomarker for pancreatic neuroendocrine tumors. HPB (Oxford). 2019;21:612–8. 30366884 10.1016/j.hpb.2018.09.016 PMC8720376

[51] Duan K Mete O. Algorithmic approach to neuroendocrine tumors in targeted biopsies: Practical applications of immunohistochemical markers. Cancer Cytopathol. 2016;124:871–84. 27529763 10.1002/cncy.21765

[52] Xu X Honda K Miura N. Actinin-4 splice variant - a complementary diagnostic and prognostic marker of pancreatic neuroendocrine neoplasms. J Cancer. 2020;11:2318–28. 32127958 10.7150/jca.37503 PMC7052930

[53] Tomita T. Significance of chromogranin A and synaptophysin in pancreatic neuroendocrine tumors. Bosn J Basic Med Sci. 2020;20:336–46. 32020844 10.17305/bjbms.2020.4632 PMC7416176

[54] Shao S Zeng Z Hu S. An observational analysis of insulinoma from a single institution. QJM. 2018;111:237–41. 29319794 10.1093/qjmed/hcy006

[55] Wiesli P Uthoff H Perren A. Are biochemical markers of neuroendocrine tumors coreleased with insulin following local calcium stimulation in patients with insulinomas? Pancreas. 2011;40:995–9. 21705951 10.1097/MPA.0b013e31821ffce1

[56] Qiao XW Qiu L Chen YJ. Chromogranin A is a reliable serum diagnostic biomarker for pancreatic neuroendocrine tumors but not for insulinomas. BMC Endocr Disord. 2014;14:64. 25099181 10.1186/1472-6823-14-64 PMC4130880

[57] Rehfeld JF Bardram L Hilsted L. An evaluation of chromogranin A versus gastrin and progastrin in gastrinoma diagnosis and control. Biomark Med. 2014;8:571–80. 24796622 10.2217/bmm.13.161

[58] Bocchini M Nicolini F Severi S. Biomarkers for pancreatic neuroendocrine neoplasms (PanNENs) management-an updated review. Front Oncol. 2020;10:831. 32537434 10.3389/fonc.2020.00831 PMC7267066

[59] Oberg K. Circulating biomarkers in gastroenteropancreatic neuroendocrine tumours. Endocr Relat Cancer. 2011;18(Suppl 1):S17–25. 22005113 10.1530/ERC-10-0280

[60] Amiri FS. Prevalence of diagnostic methods and treatment modalities in vipoma patients: a rare cause of hormone-mediated diarrhea. Indian J Endocrinol Metab. 2019;23:318–25. 31641634 10.4103/ijem.IJEM_105_19 PMC6683692

[61] Sansone A Lauretta R Vottari S. Specific and non-specific biomarkers in neuroendocrine gastroenteropancreatic tumors. Cancers (Basel). 2019;11:1113. 31382663 10.3390/cancers11081113 PMC6721814

[62] Kanakis G Kaltsas G. Biochemical markers for gastroenteropancreatic neuroendocrine tumours (GEP-NETs). Best Pract Res Clin Gastroenterol. 2012;26:791–802. 23582919 10.1016/j.bpg.2012.12.006

[63] Martin CS Parfeni OD Popa LG. How many times can one go back to the drawing board before the accurate diagnosis and surgical treatment of glucagonoma? Diagnostics (Basel). 2022;12:216. 35054383 10.3390/diagnostics12010216 PMC8774529

[64] Martin S Fica S Parfeni O. Somatostatinoma and neurofibromatosis type 1-A case report and review of the literature. Diagnostics (Basel). 2020;10:620. 32825782 10.3390/diagnostics10090620 PMC7555390

[65] Hamilton NA Liu TC Cavatiao A. Ki-67 predicts disease recurrence and poor prognosis in pancreatic neuroendocrine neoplasms. Surgery. 2012;152:107–13. 22503317 10.1016/j.surg.2012.02.011 PMC3377849

[66] Ma ZY Gong YF Zhuang HK. Pancreatic neuroendocrine tumors: A review of serum biomarkers, staging, and management. World J Gastroenterol. 2020;26:2305–22. 32476795 10.3748/wjg.v26.i19.2305 PMC7243647

[67] Reid MD Bagci P Ohike N. Calculation of the Ki67 index in pancreatic neuroendocrine tumors: a comparative analysis of four counting methodologies [published correction appears in Mod Pathol 2016 Jan;29(1):93]. Mod Pathol. 2015;28:686–94. 25412850 10.1038/modpathol.2014.156 PMC4460192

[68] Kim D Viswanathan K Goyal A. Insulinoma-associated protein 1 (INSM1) is a robust marker for identifying and grading pancreatic neuroendocrine tumors. Cancer Cytopathol. 2020;128:269–77. 31977134 10.1002/cncy.22242

[69] Villani V Mahadevan KK Ligorio M. Phosphorylated Histone H3 (PHH3) is a superior proliferation marker for prognosis of pancreatic neuroendocrine tumors. Ann Surg Oncol. 2016;23(Suppl 5):609–17. 27020585 10.1245/s10434-016-5171-x PMC8713440

[70] Lee DW Kim MK Kim HG. Diagnosis of pancreatic neuroendocrine tumors. Clin Endosc. 2017;50:537–45. 29207856 10.5946/ce.2017.131 PMC5719919

[71] Ro C Chai W Yu VE. Pancreatic neuroendocrine tumors: biology, diagnosis,and treatment. Chin J Cancer. 2013;32:312–24. 23237225 10.5732/cjc.012.10295 PMC3845620

[72] Ro C Chai W Yu VE. Pancreatic neuroendocrine tumors: biology, diagnosis,and treatment. Chin J Cancer. 2013;32:312–24. 23237225 10.5732/cjc.012.10295 PMC3845620

[73] Kanji ZS Gallinger S. Diagnosis and management of pancreatic cancer. CMAJ. 2013;185:1219–26. 23610017 10.1503/cmaj.121368 PMC3787168

[74] Falconi M Eriksson B Kaltsas G. ENETS consensus guidelines update for the management of patients with functional pancreatic neuroendocrine tumors and non-functional pancreatic neuroendocrine tumors. Neuroendocrinology. 2016;103:153–71. 26742109 10.1159/000443171 PMC4849884

[75] Hensley ML Hagerty KL Kewalramani T. American Society of Clinical Oncology 2008 clinical practice guideline update: use of chemotherapy and radiation therapy protectants. J Clin Oncol. 2009;27:127–45. 19018081 10.1200/JCO.2008.17.2627

[76] Pavel M Öberg K Falconi M. Gastroenteropancreatic neuroendocrine neoplasms: ESMO Clinical Practice Guidelines for diagnosis, treatment and follow-up. Ann Oncol. 2020;31:844–60. 32272208 10.1016/j.annonc.2020.03.304

[77] Overview: Pancreatic cancer in adults: Diagnosis and management: Guidance. NICE. Accessed August 7, 2023. https://www.nice.org.uk/guidance/ng85. 29505215

[78] Masui T Ito T Komoto I. JNETS Project Study Group Recent epidemiology of patients with gastro-entero-pancreatic neuroendocrine neoplasms (GEP-NEN) in Japan: a population-based study. BMC Cancer. 2020;20:1104. 33189127 10.1186/s12885-020-07581-y PMC7666508

[79] Wu WM Chen J Bai CM. *Zhonghua Wai Ke Za Zhi*. The Chinese guidelines for the diagnosis and treatment of pancreatic neuroendocrine neoplasms (2020). J Pancreatol. 2021;59:401–21. 10.3760/cma.j.cn112139-20210319-00135 34102722

[80] Hofland J Falconi M Christ E. European neuroendocrine tumor society (ENETS) 2023 guidance paper for functioning pancreatic neuroendocrine tumour syndromes. J Neuroendocrinol. 2023;35. 10.1111/jne.13318 37578384

[81] Kulke MH Ou FS Niedzwiecki D. Everolimus with or without bevacizumab in advanced pNET: CALGB 80701 (Alliance). Endocr Relat Cancer. 2022;29:335–44. 35324465 10.1530/ERC-21-0239 PMC9257687

[82] Yao JC Shah MH Ito T. Everolimus for advanced pancreatic neuroendocrine tumors. N Engl J Med. 2011;364:514–23. 21306238 10.1056/NEJMoa1009290 PMC4208619

[83] Ren B Rose JB Liu Y. Heterogeneity of vascular endothelial cells, De Novo arteriogenesis and therapeutic implications in pancreatic neuroendocrine tumors. J Clin Med. 2019;8:1980. 31739580 10.3390/jcm8111980 PMC6912347

[84] Lahner H Rinke A Unger N. Sunitinib efficacy in patients with advanced pNET in clinical practice. Horm Metab Res. 2016;48:575–80. 27101094 10.1055/s-0042-105289

[85] Wiedmann MW Mössner J. Safety and efficacy of sunitinib in patients with unresectable pancreatic neuroendocrine tumors. Clin Med Insights Oncol. 2012;6:381–93. 23226079 10.4137/CMO.S7350 PMC3511053

[86] Iams WT Lovly CM. Molecular pathways: clinical applications and future direction of insulin-like growth factor-1 receptor pathway blockade. Clin Cancer Res. 2015;21:4270–7. 26429980 10.1158/1078-0432.CCR-14-2518 PMC4593065

[87] Molina AM Feldman DR Voss MH. Phase 1 trial of everolimus plus sunitinib in patients with metastatic renal cell carcinoma. Cancer. 2012;118:1868–76. 21898375 10.1002/cncr.26429 PMC3609026

[88] Pusceddu S De Braud F Lo Russo G. How do the results of the RADIANT trials impact on the management of NET patients? A systematic review of published studies. Oncotarget. 2016;7:44841–7. 27057638 10.18632/oncotarget.8601 PMC5190138

[89] Sharma J Duque M Saif MW. Emerging therapies and latest development in the treatment of unresectable pancreatic neuroendocrine tumors: an update for clinicians. Therap Adv Gastroenterol. 2013;6:474–90. 10.1177/1756283X13498808 PMC3808571 24179483

[90] Luo S Wang J Wu L. Epidemiological trends for functional pancreatic neuroendocrine tumors: a study combining multiple imputation with age adjustment. Front Endocrinol (Lausanne). 2023;14:1123642. 37113484 10.3389/fendo.2023.1123642 PMC10126336

[91] Yang M Ke NW Zhang Y. Functional and non-functional pancreatic neuroendocrine tumours: ENETS or AJCC TNM staging system? Oncotarget. 2017;8:82784–95. 29137302 10.18632/oncotarget.20007 PMC5669928

[92] Jonkers YM Claessen SM Perren A. DNA copy number status is a powerful predictor of poor survival in endocrine pancreatic tumor patients. Endocr Relat Cancer. 2007;14:769–79. 17914106 10.1677/ERC-07-0111

[93] Jensen RT Niederle B Mitry E. Gastrinoma (duodenal and pancreatic). Neuroendocrinology. 2006;84:173–82. 17312377 10.1159/000098009

[94] Madoff DC Gupta S Ahrar K. Update on the management of neuroendocrine hepatic metastases. J Vasc Interv Radiol. 2006;17:1235–49; quiz 1250. 16923972 10.1097/01.RVI.0000232177.57950.71

[95] Raymond E Dahan L Raoul JL. Sunitinib malate for the treatment of pancreatic neuroendocrine tumors [published correction appears in N Engl J Med 2011 Mar 17;364(11):1082]. N Engl J Med. 2011;364:501–13. 21306237 10.1056/NEJMoa1003825

[96] Rinke A Wittenberg M Schade-Brittinger C. Placebo-Controlled, double-blind, prospective, randomized study on the effect of octreotide LAR in the control of tumor growth in patients with metastatic neuroendocrine midgut tumors (PROMID): results of long-term survival. Neuroendocrinology. 2017;104:26–32. 26731483 10.1159/000443612

[97] Wermers RA Fatourechi V Kvols LK. Clinical spectrum of hyperglucagonemia associated with malignant neuroendocrine tumors. Mayo Clin Proc. 1996;71:1030–8. 8917287 10.4065/71.11.1030

[98] Norton JA Fraker DL Alexander HR. Surgery increases survival in patients with gastrinoma. Ann Surg. 2006;244:410–9. 16926567 10.1097/01.sla.0000234802.44320.a5 PMC1856542

[99] Sada A Glasgow AE Vella A. Malignant Insulinoma: a Rare Form of Neuroendocrine Tumor. World J Surg. 2020;44:2288–94. 32128613 10.1007/s00268-020-05445-x PMC7678752

[100] Madeira I Terris B Voss M. Prognostic factors in patients with endocrine tumours of the duodenopancreatic area. Gut. 1998;43:422–7. 9863490 10.1136/gut.43.3.422 PMC1727238

[101] Chastain MA. The glucagonoma syndrome: a review of its features and discussion of new perspectives. Am J Med Sci. 2001;321:306–20. 11370794 10.1097/00000441-200105000-00003

[102] Norton JA. Surgical treatment and prognosis of gastrinoma. Best Pract Res Clin Gastroenterol. 2005;19:799–805. 16253901 10.1016/j.bpg.2005.05.003

[103] Williamson JM Thorn CC Spalding D. Pancreatic and peripancreatic somatostatinomas. Ann R Coll Surg Engl. 2011;93:356–60. 21943457 10.1308/003588411X582681 PMC3365451

